# Disparities in Video-Based Primary Care Use Among Veterans with Cardiovascular Disease

**DOI:** 10.1007/s11606-023-08475-y

**Published:** 2024-01-22

**Authors:** Rebecca Tisdale, Claudia Der-Martirosian, Caroline Yoo, Karen Chu, Donna Zulman, Lucinda Leung

**Affiliations:** 1 Veterans Affairs Palo Alto Healthcare System/Center for Innovation to Implementation (Ci2i), Palo Alto, CA USA; 2 Department of Medicine, Division of Primary Care and Population Health, Stanford University School of Medicine, Stanford, CA USA; 3 Veterans Affairs Greater Los Angeles Healthcare System/Center for the Study of Healthcare Innovation, Implementation, & Policy (CSHIIP), Los Angeles, CA USA; 4 Veterans Emergency Management Evaluation Center (VEMEC), North Hills, CA USA; 5 Department of Medicine, Division of General Internal Medicine & Health Services Research, UCLA David Geffen School of Medicine, Los Angeles, CA USA

**Keywords:** cardiovascular disease, virtual care, telehealth, primary care

## Abstract

**Background:**

Cardiovascular disease (CVD) is prevalent among Veterans, and video care enhances access to CVD care. However, it is unknown which patients with CVD conditions receive video care in primary care clinics, where a large proportion of CVD services is delivered.

**Objective:**

Characterize use of VA video primary care for Veterans with two common CVDs, heart failure and hypertension.

**Design:**

Retrospective cohort study.

**Patients:**

Veterans seen in VA primary care with diagnoses of heart failure and/or hypertension in the year prior to the COVID-19 pandemic and for the first two pandemic-years.

**Main Measures:**

The primary outcome was use of any video-based primary care visits. Using multilevel regressions, we examined the association between video care use and patient sociodemographic and clinical characteristics, controlling for time and adjusting for patient- and site-level clustering.

**Key Results:**

Of 3.8M Veterans with 51.9M primary care visits, 456,901 Veterans had heart failure and hypertension, 50,753 had heart failure only, and 3,300,166 had hypertension only. Veterans with heart failure and hypertension had an average age of 71.6 years. 2.9% were female, and 34.8% lived in rural settings. Patients who were male, aged 75 or older, or rural-dwelling had lower odds of using video care than female patients, 18–44-year-olds, and urban-dwellers, respectively (male patients’ adjusted odds ratio [AOR] 0.73, 95% confidence interval [CI] 0.72–0.74; 75 years or older, AOR 0.38, 95% CI 0.37–0.38; rural-dwellers, AOR 0.71, 95% CI 0.70–0.71). Veterans with heart failure had higher odds of video care use than those with hypertension only (AOR 1.05, 95% CI 1.04–1.06).

**Conclusions:**

Given lower odds of video primary care use among some patient groups, continued expansion of video care could make CVD services increasingly inequitable. These insights can inform equitable triage of patients, for example by identifying patients who may benefit from additional support to use virtual care.

**Supplementary Information:**

The online version contains supplementary material available at 10.1007/s11606-023-08475-y.

## INTRODUCTION

Though the Veteran Health Administration (VHA) was a leader in virtual care adoption prior to the novel coronavirus (COVID-19) pandemic, the pandemic’s onset greatly increased the use of virtual care and video care in particular. Virtual care, including video care, represents a potential solution to access issues facing VHA, and video care use has continued in VHA even after the resolution of the most acute phase of the pandemic.^1, 2^

It is particularly important to understand the implications of virtual care for cardiovascular disease (CVD), given the general public health importance of CVD as the most common cause of death and most prevalent chronic conditions for Americans.^3, 4^ While the evidence base for effectiveness of virtual care is relatively nascent, much of the evidence that does exist for effective virtual care use—and particularly video care use—pertains to chronic disease care,^5^ meaning chronic CVD conditions may be a good fit for virtual care delivery.

CVD is frequently managed in primary care settings;^6, 7^ as access to specialty cardiology care is often limited by supply,^8^ especially in rural areas,^9^ maximizing primary care capacity to provide this CVD care is critical. Given the benefits of telehealth for both access^10, 11^ and patient satisfaction,^12^ access to this CVD-focused primary care via virtual care is valuable for patients and clinicians. Yet most studies of telehealth for CVD, particularly in the COVID-19 era, take place in the specialty cardiology setting.^2, 13–15^ By their nature, these specialty care–focused studies have captured a relatively narrow swath of telehealth use for CVD. This study, conversely, examines telehealth use for patients with CVD at the population level via primary care rather than specialty care visits.

Veterans with CVD represent a group with particularly high care needs; prevalence of cardiovascular disease among Veterans is disproportionately high,^16^ and meeting these patients’ access needs is likely to require ongoing use of virtual care as alternative solutions, such as transporting patients to more distant clinics, hiring additional specialists, or routing patients to community care, can be costly.^17^ The VHA’s strong infrastructure for telehealth,^1, 18–22^ primary care, and research coupled with the high need for CVD care among Veterans makes this an ideal setting to study virtual CVD care.

As the literature describing Veterans’ use of video care has matured, certain groups have consistently been found to use less video care than others; for other characteristics, there is no such consensus. For example, older Veterans have consistently used less video care than younger individuals, and rural-dwelling Veterans have used less video care than urban-dwellers.^2, 18, 19^ Conversely, female Veterans have been found to use more video care than male Veterans in primary care,^1, 18, 19^ but not in specialty cardiology care.^2^ There is even more variability in the degree and direction of differences in video care use by patient race/ethnicity.^23–26^ Differences by clinical condition are still less characterized: while common chronic CVD conditions like hypertension and heart failure are generally thought to be appropriate for virtual care,^27, 28^ studies of video care use among patients with these conditions are limited.^15, 29^

This study attempts to address this CVD condition evidence gap, taking into account those characteristics found to affect video care use and those for which results have been mixed. We focus on video visits and exclude other telehealth modalities, such as phone visits, because literature suggests larger disparities in video visits^1, 2, 18, 19^ and phone visits tend to be more heterogeneous in purpose and intensity. In choosing patient characteristics to include in analytical models, we draw on the literature referenced above as well as Fortney et al.’s conceptualization of healthcare access for the twenty-first century.^30^ This model posits five dimensions of access barriers, onto which we have mapped the majority of included covariates: geographic (patient rurality and drive time); temporal (pandemic-year); financial (VA enrollment priority group); cultural (patient race/ethnicity); and digital (broadband access). We focus on two cardiovascular conditions, heart failure and hypertension; these are both prevalent^4^ and require chronic management and collection of vital sign and other physical exam data, raising questions about how well matched they are for virtual care. For example, in the context of a virtual visit, can a clinician safely forgo auscultation of the patient’s heart and visual inspection of neck veins and lower extremities–maneuvers generally deemed central to the examination of a patient with heart failure?^31^

In this study, we examine changes in video-based primary care use over time for Veterans with heart failure and/or hypertension, and which patient sub-populations were likelier to access video care.

## METHODS

### Data

We used electronic health record data from VHA’s Corporate Data Warehouse repository from 3/16/2019, 1 year prior to the onset of the COVID-19 pandemic,^32^ through 3/15/2022, 2 years after the pandemic’s onset. We identified all Veterans in the USA with either a diagnosis for heart failure and/or hypertension coded at a primary care visit during this 3-year period (see Supplemental Table 1 for included International Classification of Disease-10 [ICD10] codes). We then included all primary care visits during the study period for these Veterans.

The primary outcome was the odds of ever having a video primary care visit. We categorized Veterans’ use of primary care video visits (“VVC,” for “VA Video Connect,” VA’s video-to-home visit platform) into VVC non-users (0 VVC) or users (1 or more VVC) for each year of the study. As in prior work, we used VHA-specific codes corresponding to video-based primary care to identify these visits.^33^

We included patient-level sociodemographic and clinical characteristics. These included age at the pandemic’s onset; birth sex; race/ethnicity; marital status; rurality; and Charlson Comorbidity Index (CCI), a measure of chronic conditions predicting risk of future mortality.^34^ While we do not have income reported by Veterans, we included VHA enrollment priority grouping, which integrates information generated from an income verification procedure (means test) and thus may approximate socioeconomic vulnerability;^35, 36^ detailed information on the above variables is included in the Supplement. The site where the majority of a patient’s primary care visits took place (or in the case of ties, the site of most recent primary care visit) was captured as a VHA healthcare system, or VHA hospital and surrounding clinics (*N*=138).

### Data Analysis

After generating descriptive statistics for the number of video visits by each year, we constructed a two-level mixed effects multivariable logistic regression model adjusted for patient clinical and sociodemographic characteristics to estimate the association between patient characteristics and the primary outcome, odds of ever using video primary care in a given year. Models controlled for time and adjusted for patient- and site-level clustering (i.e., patient and site represent the two levels in the two-level mixed model); site was the VHA healthcare system or VHA hospital and surrounding clinics described above. In addition to this baseline model, we performed sensitivity analyses with models that included interactions between patient variables found or hypothesized to have significant interactions (e.g., age and sex,^1^ age and race, race and rurality), as well as a sensitivity analysis including additional sociodemographic characteristics that can affect access to care: drive distance from the patient’s residence to the nearest primary care site,^37^ captured as a distance less than or equal to versus greater than 40 miles; patient broadband access,^38^ defined as having at least one fixed Internet provider at speed 25/3 Mbps in patient residence’ census block and available for a subset of patients; and facility complexity^38^ of the patient’s usual VA hospital.

Data analysis was performed using Stata 17 (StataCorp LLC, College Station, TX). This study was part of an ongoing VA quality improvement effort approved by the VA Greater Los Angeles Healthcare System institutional review board as non-human subjects research and, therefore, exempt from informed consent requirements, and follows the Strengthening the Reporting of Observational Studies in Epidemiology (STROBE) reporting guidelines.^39^

## RESULTS

**Figure 1:**
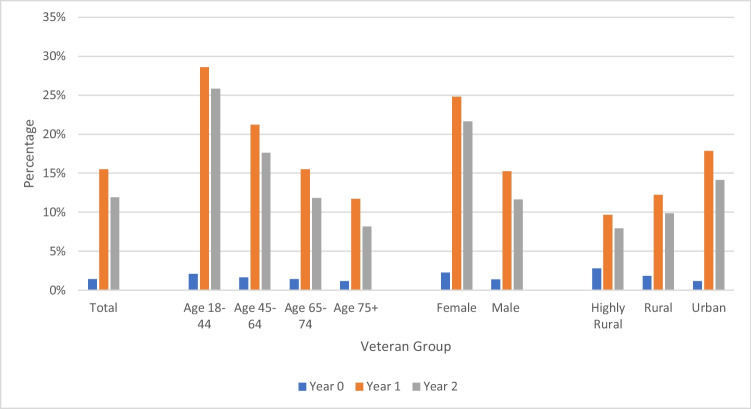
Veterans with heart failure and hypertension with 1+ video primary care visit (unadjusted), 3/2019–3/2022.

Our analytic cohort comprised 3,807,820 Veterans with diagnoses of heart failure, hypertension, or both, with 51,878,099 total primary care visits over the 3-year study period. Veterans with both heart failure and hypertension (*n*=456,901; Table 1) had an average baseline age of 71.6 years and mean CCI of 3.0, 2.9% were female at birth, and 34.8% lived in a rural or highly rural setting. Only 1.4% of these patients had a video visit in the year prior to the pandemic, compared to 15.5% of patients with a video visit in the first year of the pandemic and 11.9% in the second year (Fig. 1). Veterans with hypertension (*n*=3,757,067; *n*=3,300,166 with hypertension and without heart failure; Supplemental Table 2) were younger and healthier on average, with a mean age of 66.7 years and mean CCI of 1.4. 5.6% of these Veterans were female at birth, and 34.7% lived in rural, insular, or highly rural settings. Use of video care was similar to use in those with both heart failure and hypertension with slightly more sustained use, with 1.4% having at least one video visit in the year prior to the pandemic, 15.8% in the pandemic’s first year, and 12.9% in the second year. The group of Veterans with heart failure (*n*=507,654; *n*=50,753 with heart failure and without hypertension; Supplemental Table 3) appeared very similar to those with heart failure and hypertension, as the majority (90%) of those with a diagnosis of heart failure also had hypertension.

**Table 1:** Baseline Patient Characteristics and Video Care Use Over Time, Veterans with Heart Failure and Hypertension (*N*=456,901). Year 0 refers to the pre-pandemic year: 3/16/2019–3/15/2020; Year 1 refers to pandemic year 1: 3/16/2020–3/15/2021; Year 2 refers to pandemic year 2: 3/16/2021–3/15/2022

	Year 0			Year 1			Year 2		
	Total	0 VVC^†^	1+ VVC	Total	0 VVC	1+ VVC	Total	0 VVC	1+ VVC
*N* (%)	456,901 (100.0)	450,547 (98.6)	6354 (1.4)	–	385,965 (84.5)	70,936 (15.5)	–	402,442 (88.1)	54,459 (11.9)
Baseline age, mean (SD)	71.6 (10.7)	71.6 (10.7)	69.9 (11.0)	–	72.1 (10.5)	68.9 (11.2)	–	72.1 (10.5)	68.1 (11.2)
Baseline age category									
18–44	5797 (1.3)	5675 (1.3)	122 (1.9)	–	4139 (1.1)	1658 (2.3)	–	4298 (1.1)	1499 (2.8)
45–64	96,024 (21.0)	94,449 (21.0)	1575 (24.8)	–	75,654 (19.6)	20,370 (28.7)	–	79,120 (19.7)	16,904 (31.0)
65–74	193,545 (42.4)	190,787 (42.4)	2758 (43.4)	–	163,528 (42.4)	30,017 (42.3)	–	170,665 (42.4)	22,880 (42.0)
75+	161,535 (35.4)	159,636 (35.4)	1899 (29.9)	–	142,644 (37.0)	18,891 (26.6)	–	148,359 (36.9)	13,176 (24.2)
Birth sex									
Female	13,405 (2.9)	13,106 (2.9)	299 (4.7)	–	10,078 (2.6)	3327 (4.7)	–	10,506 (2.6)	2899 (5.3)
Baseline race/ethnicity									
Non-Hispanic Black	86,180 (18.9)	85,242 (18.9)	938 (14.8)	–	70,918 (18.4)	15,262 (21.5)	–	73,250 (18.2)	12,930 (23.7)
Hispanic	20,618 (4.5)	20,341 (4.5)	277 (4.4)	–	16,290 (4.2)	4328 (6.1)	–	17,482 (4.3)	3136 (5.8)
Non-Hispanic other race	8254 (1.8)	8095 (1.8)	159 (2.5)	–	6862 (1.8)	1392 (2.0)	–	7178 (1.8)	1076 (2.0)
Unknown race/ethnicity	23,527 (5.2)	23,198 (5.2)	329 (5.2)	–	20,267 (5.3)	3260 (4.6)	–	20873 (5.2)	2654 (4.9)
Non-Hispanic White	31,8322 (69.7)	31,3671 (69.6)	4651 (73.2)	–	271,628 (70.4)	46,694 (65.8)	–	283,659 (70.5)	34,663 (63.7)
Enrollment priority									
High disability	169,382 (37.1)	166,761 (37.0)	2621 (41.3)	–	139,900 (36.3)	29,482 (41.6)	–	146,768 (36.5)	22,614 (41.5)
Low/moderate disability	82,544 (18.1)	81,374 (18.1)	1170 (18.4)	–	69,351 (18.0)	13,193 (18.6)	–	72,452 (18.0)	10,092 (18.5)
Low income	120,288 (26.3)	118,766 (26.4)	1522 (24.0)	–	103,987 (26.9)	16,301 (23.0)	–	107,754 (26.8)	12,534 (23.0)
No service disability	74,725 (16.4)	73,737 (16.4)	988 (15.6)	–	64,280 (16.7)	10,445 (14.7)	–	66,959 (16.6)	7766 (14.3)
Unknown	9962 (2.2)	9909 (2.2)	53 (0.8)	–	8447 (2.2)	1515 (2.1)	–	8509 (2.1)	1453 (2.7)
Marital status									
Divorced	107,380 (23.5)	105,793 (23.5)	1587 (25.0)	104,385 (22.9)	87,800 (22.8)	16,585 (23.4)	95,526 (20.9)	82,145 (20.4)	13,381 (24.6)
Married	245,432 (53.7)	241,757 (53.7)	3675 (57.8)	237,852 (52.1)	197,523 (51.2)	40,329 (56.9)	216,097 (47.3)	186,060 (46.2)	30,037 (55.2)
Never married	37,526 (8.2)	37,102 (8.2)	424 (6.7)	36,715 (8.0)	30,874 (8.0)	5841 (8.2)	33,866 (7.4)	29,040 (7.2)	4826 (8.9)
Separated	14,767 (3.2)	14,580 (3.2)	187 (2.9)	14,284 (3.1)	11,748 (3.0)	2536 (3.6)	13,108 (2.9)	11,082 (2.8)	2026 (3.7)
Widowed	38,643 (8.5)	38,203 (8.5)	440 (6.9)	37,732 (8.3)	32,551 (8.4)	5181 (7.3)	33,215 (7.3)	29,447 (7.3)	3768 (6.9)
Unknown	13,153 (2.9)	13,112 (2.9)	41 (0.7)	25,933 (5.7)	25,469 (6.6)	464 (0.7)	65,089 (14.3)	64,668 (16.1)	421 (0.8)
Rurality									
Highly rural	6077 (1.3)	5907 (1.3)	170 (2.7)	18,979 (4.2)	17,145 (4.4)	1834 (2.6)	18,106 (4.0)	16,668 (4.1)	1438 (2.6)
Insular	296 (0.1)	267 (0.1)	29 (0.5)	289 (0.1)	259 (0.1)	30 (0.04)	269 (0.1)	244 (0.1)	25 (0.1)
Rural	153,202 (33.5)	150,421 (33.4)	2781 (43.8)	141,028 (30.9)	123,776 (32.1)	17,252 (24.3)	136,038 (29.8)	122,627 (30.5)	13,411 (24.6)
Urban	286,238 (62.7)	282,939 (62.8)	3299 (51.9)	286,467 (62.7)	235,372 (61.0)	51,095 (72.0)	276,970 (60.6)	237,825 (59.1)	39,145 (71.9)
Unknown	11,088 (2.4)	11,013 (2.4)	75 (1.2)	10,138 (2.2)	9413 (2.4)	725 (1.0)	25,518 (5.6)	25,078 (6.2)	440 (0.8)
Charlson comorbidity index, mean (SD)	3.0 (2.7)	3.0 (2.7)	3.1 (2.7)	3.6 (2.9)	3.6 (2.9)	3.6 (2.8)	3.3 (2.9)	3.2 (2.9)	3.5 (2.8)
Charlson comorbidity index, *n* (%)									
0	87,908 (19.2)	86,894 (19.3)	1014 (16.0)	53,126 (11.6)	45,003 (11.7)	8123 (11.5)	78,254 (17.1)	72,128 (17.9)	6126 (11.3)
1	76,175 (16.7)	75,089 (16.7)	1086 (17.1)	67,210 (14.7)	56,755 (14.7)	10,455 (14.7)	68,083 (14.9)	59,609 (14.8)	8474 (15.6)
2+	292,818 (64.1)	288,564 (64.1)	4254 (67.0)	336,565 (73.7)	284,207 (73.6)	52,358 (73.8)	310,564 (68.0)	270,705 (67.3)	39,859 (73.2)
Driving distance to nearest primary care site									
40+ miles	30 072 (6.7)	29 431 (6.6)	641 (10.1)	30 072 (6.7)	26 967 (7.1)	3105 (4.4)	30 072 (6.7)	27 605 (6.9)	2467 (4.6)
Facility complexity									
1a—high complexity	184,286 (42.7)	181,747 (42.8)	2539 (39.9)	172,181 (43.0)	137,040 (41.6)	35,141 (49.5)	161,955 (44.8)	133,056 (43.3)	28,899 (53.0)
1b—high complexity	77,297 (17.9)	76,133 (17.9)	1164 (18.3)	71,683 (17.9)	58,442 (17.7)	13,241 (18.6)	61,608 (17.0)	52,140 (17.0)	9468 (17.3)
1c—high complexity	92,451 (21.4)	91,502 (21.5)	949 (14.9)	85,620 (21.4)	71,638 (21.7)	13,982 (19.7)	57,085 (15.8)	49,588 (16.1)	7497 (13.7)
2—medium complexity	39,056 (9.0)	38,129 (8.9)	927 (14.5)	36,182 (9.0)	31,643 (9.6)	4539 (6.4)	43,348 (12.0)	38,482 (12.5)	4866 (8.9)
3— low complexity	3754 (8.7)	36,779 (8.6)	775 (12.2)	34,314 (8.5)	30,282 (9.2)	4032 (5.6)	35,765 (9.9)	32,106 (10.4)	3659 (6.7)
98—excluded	68 (0.0)	68 (0.0)	0 (0)	56 (0.0)	55 (0.0)	1 (0)	1317 (0.3)	1247 (0.4)	70 (0.1)
Broadband access									
Yes	412,932 (92.9)	407,213 (92.9)	5719 (91.1)	412,932 (92.9)	346,119 (92.5)	66,813 (95.0)	412,932 (92.9)	362,311 (92.6)	50,621 (94.7)

^†^VVC refers to VA Video Connect, VA’s video visit platform

^‡^Cells with total N unchanged from baseline year are filled with “–” for clarity

**Figure 2:**
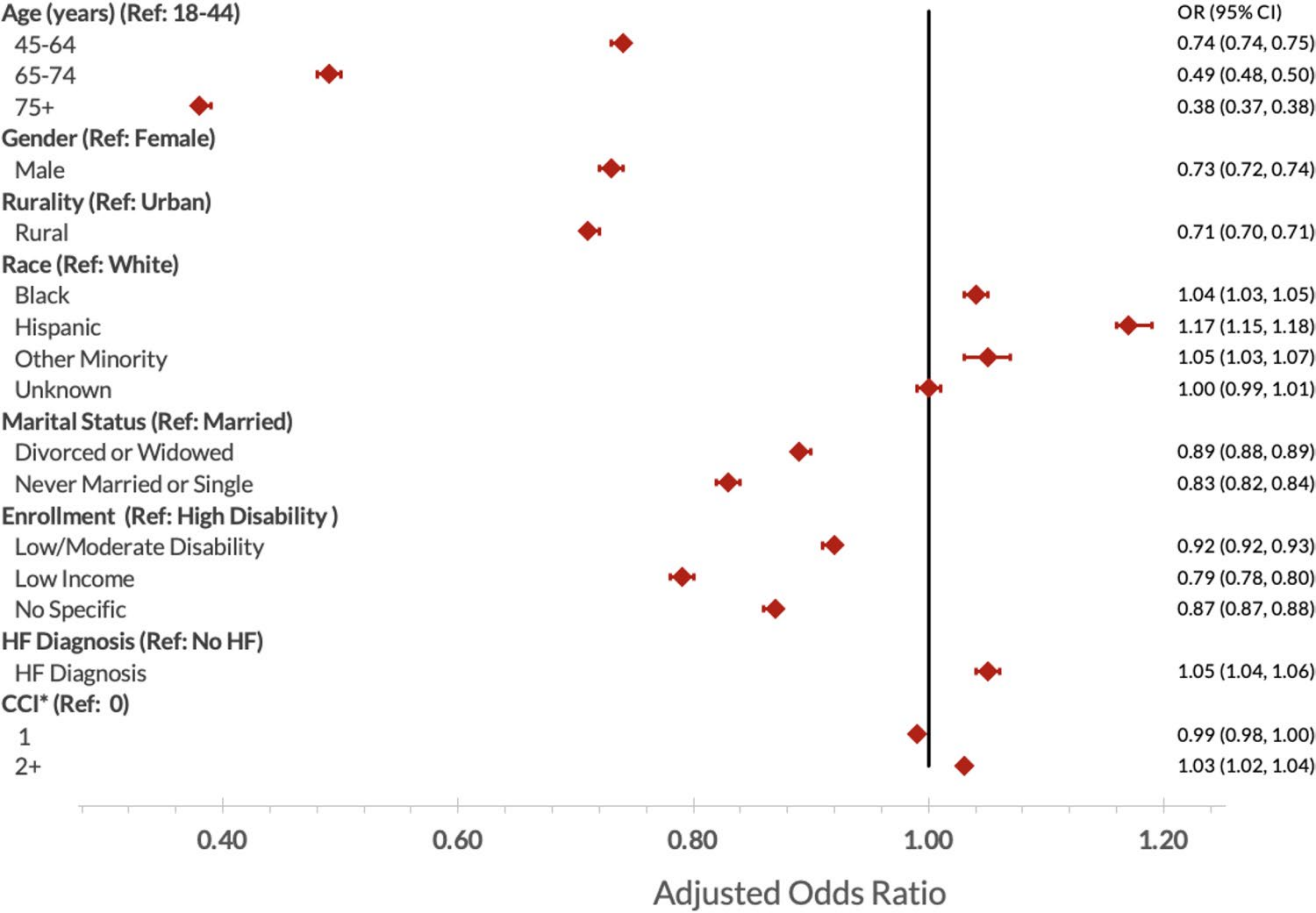
Adjusted odds of ever using video primary care among 3,807,820 Veterans with hypertension, heart failure, or both. *CCI, Charlson comorbidity index.

Trends in video primary care use among patients with CVD were the same in adjusted and unadjusted analyses. In our multi-level logistic regression model (Fig. 2, Supplemental Table 4), odds of using video primary care were highest during the first year of the pandemic, then declined in the second (AOR 15.3, 95% CI 15.1–15.4 and 11.5, 95% CI 11.3–11.6, respectively, compared to the pre-pandemic year). Male patients had lower odds of ever using video care than female patients (adjusted odds ratio [AOR] 0.73, 95% confidence interval [CI] 0.72–0.74). Older patients in each age category had lower odds of using video care than younger individuals: patients 75 years or older had an AOR of 0.38 (95% CI 0.38–0.39), those aged 65–74 had an AOR of 0.49 (95% CI 0.49–0.50), and 45–64-year-olds had an AOR of 0.75 (95% CI 0.74, 0.76) compared to those aged 18–44 years. Rural-dwelling Veterans had lower odds of using video care than urban-dwellers (AOR 0.71, 95% CI 0.70–0.71). Compared to Veterans with high disability, those in all other enrollment priority groups had lower odds of using video care; low-income Veterans had the lowest odds of using video care among these categories, with an AOR of 0.79 (95% CI 0.78–0.80). Those who were divorced or widowed or who were never married or single had lower odds of using video care compared to married Veterans (AOR 0.89 [95% CI 0.88–0.90] for divorced or widowed, 0.83 [95% CI 0.82–0.84] for never married or single). Veterans with heart failure had slightly higher odds of video care use than those with hypertension only (AOR 1.06, 95% CI 1.05–1.07). Those with more comorbidities in general (CCI of 2 or more) also had slightly higher odds of video care compared to those with a CCI of 0 (AOR 1.03, 95% CI 1.02–1.04).

In sensitivity analyses in which we included interaction terms in the model for various sociodemographic characteristics (Supplemental Table 5), the overall effects of these characteristics on the primary outcome were largely unchanged. We found a significant interaction between these characteristics for Veterans of Hispanic ethnicity (AOR for the interaction term 1.18, 95% CI 1.15,1.22) and non-Hispanic other race/ethnicity (AOR 0.88, 95% CI 0.85,0.93). This suggests that the effect of living in a rural versus urban environment varies according to race/ethnicity for these populations. However, as noted, the overall effects of race/ethnicity on the primary outcome were unchanged in this model. Results were also similar to the main model in our sensitivity analysis with additional sociodemographic characteristics (Supplemental Table 6). While drive distance greater than 40 miles was associated with lower adjusted odds of video care use (AOR 0.91, 95% CI 0.90, 0.92), the rurality variable likely captured this effect in the main model, as the rurality effect was attenuated in the sensitivity analysis. Lower facility complexity was associated with lower adjusted odds of video care use (AOR 0.89, 95% CI 0.89,0.89), whereas the converse was true for broadband access (AOR 1.17, 95% CI 1.16, 1.19).

## DISCUSSION

In this retrospective cohort analysis of Veterans with heart failure and/or hypertension, we found substantial differences in use of video primary care across patient groups corresponding to all dimensions of access set out in our conceptual model.^30^ In particular, and similar to the general population of Veterans, male, older, low-income, and rural-dwelling Veterans with these prevalent cardiovascular comorbidities had lower odds of ever using video primary care compared to their respective reference groups. Additionally, patients with heart failure had slightly higher odds of using video primary care than those without heart failure.

To the extent that there were differences by race or ethnicity in the adjusted odds of using video primary care in this study, Veterans from historically marginalized racial/ethnic groups had similar or higher odds of video use compared to White Veterans, consistent with prior studies of pandemic-era VA video primary and cardiology care.^2, 18, 19^ As racial disparities have been identified in heart failure care at every stage from diagnosis to referral for advanced heart failure therapies,^40–42^ it is encouraging that here, as in other facets of CVD care,^43^ disparities by race and ethnicity were smaller within the VHA’s integrated healthcare system than in studies conducted in other systems.^23–26^

In contrast to these minimal racial or ethnic differences, we did find evidence of disparities in video care use by age, rurality, and income. These results suggest that Veterans with CVD are similar to the general population of Veterans^18, 19^ and Veterans engaged in specialty cardiology care^2^ in that Veterans who are older, living in rural areas, or lower-income are at risk for falling on the wrong side of this digital divide.^44^ This is concerning, given that some of the benefits of video visits (e.g., time and money saved by forgoing travel) would be expected to be most salient to these individuals,^45, 46^ and suggests an opportunity for interventions as discussed below.

Perhaps in contrast to literature warning clinicians to proceed with caution when conducting virtual visits for patients with heart failure,^47^ we found slightly higher adjusted odds of video care use among Veterans with heart failure compared to those with hypertension only. This may reflect that patients with heart failure have more frequent interactions with the healthcare system on average, including via virtual modalities; virtual visits can be integrated into heart failure care workflows to optimize risk factor modification, medication adherence, and symptom monitoring.^48^ As our data do not include the particular reasons for a given patient visit (i.e., these are visits among patients with CVD, and for whom at least one visit in the study period pertained to their CVD, but not necessarily *for* CVD at each visit), we could not draw conclusions about the modality of heart failure-related visits specifically. Still, these results suggest that at a minimum, patients with heart failure are using video care as much or more than patients with hypertension only.

### Potential Solutions

While highlighting disparities in care delivery is a necessary first step for improving health equity, this process is only useful insofar as solutions are identified and implemented. VA’s tablet distribution initiative is one such effort to expand access to individuals who might otherwise face digital divide disparities.^49, 50^ Other federal initiatives include Lifeline^51^ and the Affordable Connectivity Program,^52^ which subsidize internet and/or phone services and Internet-enabled device purchases. Outside the government, organizations like EveryoneOn work to connect lower-income people living in the USA to low-cost home internet service and affordable devices.^53^ Virtual models of care that are designed to meet the needs of rural-dwelling, geriatric, and other historically underserved populations, such as VA’s Clinical Resource Hub (CRH) program, have also been shown to mitigate the digital divide.^10^ Such programs moderate the risk of growing disparities as more care is delivered virtually, though further research is needed to establish whether they are sufficient to meet this goal as currently implemented.^50, 54^

### Limitations

The goal of this study was to characterize use of primary care video visits for this population; future analyses should examine clinical care delivery through the combination of video visits and phone visits, given that phone visits comprise a large proportion of VHA virtual care.^2, 18, 19^ Secondly, the current study design and data prevent us from identifying provider-level variation in video care delivery, which can impact patient use of such services.

Our study also has limitations common to observational studies in the VHA, including generalizability to the general US population, though the national scope and large sample size represent strengths of this VHA system focus. As in most telehealth research, there is a risk of visit modality misclassification, since the electronic medical record does not reliably capture when modality changes during a visit (e.g., a video visit is converted to a phone visit due to technologic issues). However, conversion from video to phone visits occurs disproportionately among some of the groups found to use video care less often,^55^ meaning the differences identified are more likely to be underestimates than overestimates. Finally, our inclusion of only patients with at least one primary diagnostic code for heart failure or hypertension minimized the risk of including patients without these conditions of interest, but may have inadvertently excluded some patients with these conditions.

## Conclusion

With the onset of the COVID-19 pandemic, video care became and remains an important proportion of primary care delivered to Veterans with cardiovascular disease. Given lower odds of video care among certain Veteran groups, continued expansion of video care could make CVD services increasingly inequitable unless solutions are implemented simultaneously. As VA expands virtual care for CVD, these insights can inform equitable and effective triage of patients to virtual versus in-person care by identifying patients that may require additional support to use virtual care.

### Supplementary Information

Below is the link to the electronic supplementary material.

Supplementary file1 (DOCX 56 KB)

## Data Availability

Access to datasets generated during and/or analyzed during the current study is not available per government policy, but relevant code may be provided to researchers within the Veterans Administration (VA) upon request.

## References

[1] Der-Martirosian C, Chu K, Steers WN (2022). Examining telehealth use among primary care patients, providers, and clinics during the COVID-19 pandemic. BMC Prim Care.

[2] **Tisdale RL, Ferguson J, Van Campen J, et al.** Disparities in virtual cardiology visits among Veterans Health Administration patients during the COVID-19 pandemic. JAMIA Open 2022;5(4):ooac103. 10.1093/jamiaopen/ooac103 PMC9754629 36531138

[3] **Weir HK, Anderson RN, King SMC, et al.** Heart Disease and Cancer Deaths — Trends and Projections in the United States, 1969–2020. Prev Chronic Dis 2016;13. 10.5888/pcd13.160211 PMC5127176 27854420

[4] Tsao CW, Aday AW, Almarzooq ZI (2022). Heart Disease and Stroke Statistics—2022 Update: A Report From the American Heart Association. Circulation.

[5] **Totten AM, Hansen RN, Wagner J, et al.** Telehealth for Acute and Chronic Care Consultations [Internet]. Agency for Healthcare Research and Quality; 2019 [cited 2023 Mar 27]. Available from: https://effectivehealthcare.ahrq.gov/topics/telehealth-acute-chronic/research

[6] Cleland J, Cohen-Solal A, Aguilar JC (2002). Management of heart failure in primary care (the IMPROVEMENT of Heart Failure Programme): an international survey. The Lancet.

[7] Jones NR, Hobbs FDR, Taylor CJ (2017). The management of diagnosed heart failure in older people in primary care. Maturitas.

[8] Griffith KN, Ndugga NJ, Pizer SD (2020). Appointment Wait Times for Specialty Care in Veterans Health Administration Facilities vs Community Medical Centers. JAMA Netw Open.

[9] Barreto T, Jetty A, Eden AR, Petterson S, Bazemore A, Peterson LE (2021). Distribution of Physician Specialties by Rurality. J Rural Health.

[10] **Leung LB, Rubenstein LV, Jaske E, Wheat CL, Nelson KM, Felker BL.** Contrasting Care Delivery Modalities Used by Primary Care and Mental Health Specialties in VA’s Telehealth Contingency Staffing Program During the COVID-19 Pandemic. J Gen Intern Med [Internet] 2022 [cited 2022 Jul 14];Available from: 10.1007/s11606-022-07527-z 10.1007/s11606-022-07527-z PMC9017959 35441301

[11] Cannedy S, Bergman A, Medich M, Rose DE, Stockdale SE (2022). Health System Resiliency and the COVID-19 Pandemic: A Case Study of a New Nationwide Contingency Staffing Program. Healthcare.

[12] Olayiwola JN, Magaña C, Harmon A (2020). Telehealth as a Bright Spot of the COVID-19 Pandemic: Recommendations From the Virtual Frontlines (“Frontweb”). JMIR Public Health Surveill.

[13] Mishra K, Edwards B (2022). Cardiac Outpatient Care in a Digital Age: Remote Cardiology Clinic Visits in the Era of COVID-19. Curr Cardiol Rep.

[14] Yuan N, Pevnick JM, Botting PG (2021). Patient Use and Clinical Practice Patterns of Remote Cardiology Clinic Visits in the Era of COVID-19. JAMA Netw Open.

[15] **Kalwani NM, Osmanlliu E, Parameswaran V, et al. **Changes in telemedicine use and ambulatory visit volumes at a multispecialty cardiovascular center during the COVID-19 pandemic. J Telemed Telecare 2022;1357633X211073428. 10.1177/1357633X211073428 PMC8814611 35108126

[16] Hinojosa R (2019). Veterans’ Likelihood of Reporting Cardiovascular Disease. J Am Board Fam Med.

[17] **Burkhardt JE, Rubino J, Yum J.** Improving Mobility for Veterans [Internet]. The National Academies Press; [cited 2023 Jun 5]. Available from: https://nap.nationalacademies.org/download/14507#

[18] **Ferguson JM, Jacobs J, Yefimova M, Greene L, Heyworth L, Zulman DM.** Virtual care expansion in the Veterans Health Administration during the COVID-19 pandemic: clinical services and patient characteristics associated with utilization. J Am Med Inform Assoc 2020;ocaa284. 10.1093/jamia/ocaa284 PMC7665538 33125032

[19] **Ferguson JM, Wray CM, Jacobs J, et al.** Variation in initial and continued use of primary, mental health, and specialty video care among Veterans. Health Serv Res [Internet] [cited 2023 Jan 6];n/a(n/a). Available from: https://onlinelibrary.wiley.com/doi/abs/10.1111/1475-6773.14098 10.1111/1475-6773.14098 PMC10012228 36345235

[20] **Heyworth L, Kirsh S, Zulman D, Ferguson JM, Kizer KW**. Expanding Access through Virtual Care: The VA’s Early Experience with Covid-19. NEJM Catal 2020;11.

[21] **Der-Martirosian C, Chu K, Dobalian A**. Use of Telehealth to Improve Access to Care at the United States Department of Veterans Affairs During the 2017 Atlantic Hurricane Season. Disaster Med Public Health Prep undefined/ed;1–5. 10.1017/dmp.2020.88 32279689

[22] Reddy A, Gunnink E, Deeds SA (2020). A rapid mobilization of ‘virtual’ primary care services in response to COVID-19 at Veterans Health Administration. Healthcare.

[23] **Rodriguez JA, Betancourt JR, Sequist TD, Ganguli I.** Differences in the Use of Telephone and Video Telemedicine Visits During the COVID-19 Pandemic. 2021 [cited 2023 Mar 28];27. Available from: https://www.ajmc.com/view/differences-in-the-use-of-telephone-and-video-telemedicine-visits-during-the-covid-19-pandemic 10.37765/ajmc.2021.88573 PMC10877492 33471458

[24] Eberly LA, Khatana SAM, Nathan AS (2020). Telemedicine Outpatient Cardiovascular Care During the COVID-19 Pandemic: Bridging or Opening the Digital Divide?. Circulation.

[25] Reed ME, Huang J, Graetz I (2020). Patient Characteristics Associated With Choosing a Telemedicine Visit vs Office Visit With the Same Primary Care Clinicians. JAMA Netw Open.

[26] Weber E, Miller SJ, Astha V, Janevic T, Benn E (2020). Characteristics of telehealth users in NYC for COVID-related care during the coronavirus pandemic. J Am Med Inform Assoc.

[27] Gorodeski EZ, Goyal P, Cox ZL (2020). Virtual Visits for Care of Patients with Heart Failure in the Era of COVID-19: A Statement from the Heart Failure Society of America. J Card Fail.

[28] Kalagara R, Chennareddy S, Scaggiante J (2022). Blood pressure management through application-based telehealth platforms: a systematic review and meta-analysis. J Hypertens.

[29] **Koos H, Parameswaran V, Claire S, et al. **Drivers of variation in telemedicine use during the COVID-19 pandemic: The experience of a large academic cardiovascular practice. J Telemed Telecare 2022;1357633X221130288. 10.1177/1357633X221130288 PMC9549164 36214200

[30] Fortney JC, Burgess JF, Bosworth HB, Booth BM, Kaboli PJ (2011). A Re-conceptualization of Access for 21st Century Healthcare. J Gen Intern Med.

[31] **Mann DL, Chakinala M**. Heart Failure: Pathophysiology and Diagnosis [Internet]. In: **Jameson JL, Fauci AS, Kasper DL, Hauser SL, Longo DL, Loscalzo J, editors.** Harrison’s Principles of Internal Medicine. New York, NY: McGraw-Hill Education; 2018 [cited 2022 May 5]. Available from: accessmedicine.mhmedical.com/content.aspx?aid=1173780154

[32] World Health Organization. WHO Director-General’s opening remarks at the media briefing on COVID-19 - 11 March 2020 [Internet]. [cited 2021 Apr 20];Available from: https://www.who.int/director-general/speeches/detail/who-director-general-s-opening-remarks-at-the-media-briefing-on-covid-19---11-march-2020

[33] Leung LB, Yoo C, Chu K (2023). Rates of Primary Care and Integrated Mental Health Telemedicine Visits Between Rural and Urban Veterans Affairs Beneficiaries Before and After the Onset of the COVID-19 Pandemic. JAMA Netw Open.

[34] Charlson ME, Pompei P, Ales KL, MacKenzie CR (1987). A new method of classifying prognostic comorbidity in longitudinal studies: Development and validation. J Chronic Dis.

[35] Wang ZJ, Dhanireddy P, Prince C, Larsen M, Schimpf M, Pearman G (2019). Survey of Veteran Enrollees’ Health and Use of Health Care..

[36] VA priority groups [Internet]. Veterans Aff. 2022 [cited 2022 Oct 14];Available from: https://www.va.gov/health-care/eligibility/priority-groups/

[37] Jazowski SA, Sico IP, Lindquist JH (2021). Transportation as a barrier to colorectal cancer care. BMC Health Serv Res.

[38] **Jacobs J, Ferguson JM, Van Campen J, et al.** Organizational and External Factors Associated with Video Telehealth Use in the Veterans Health Administration Before and During the COVID-19 Pandemic. Telemed E-Health 2021; 10.1089/tmj.2020.0530 33887166

[39] **STROBE I.** The Strengthening the Reporting of Observational Studies in Epidemiology (STROBE) statement: guidelines for reporting observational studies. [cited 2021 Apr 20];Available from: https://core.ac.uk/reader/33050540?utm_source=linkout 10.1136/bmj.39335.541782.AD PMC2034723 17947786

[40] **Virani SS, Alonso A, Benjamin EJ, et al.** Heart Disease and Stroke Statistics—2020 Update: A Report From the American Heart Association. Circulation [Internet] 2020 [cited 2020 Nov 25];141(9). Available from: https://www.ahajournals.org/doi/10.1161/CIR.0000000000000757 10.1161/CIR.0000000000000757 31992061

[41] Breathett K, Liu WG, Allen LA (2018). African Americans Are Less Likely to Receive Care by a Cardiologist During an Intensive Care Unit Admission for Heart Failure. JACC Heart Fail.

[42] Breathett K, Allen LA, Helmkamp L (2018). Temporal Trends in Contemporary Use of Ventricular Assist Devices by Race and Ethnicity. Circ Heart Fail.

[43] **Tisdale RL, Fan J, Calma J, et al.** Predictors of Incident Heart Failure Diagnosis Setting: Insights from the Veterans Affairs Healthcare System. 10.1016/j.jchf.2022.11.013 PMC10069381 36881392

[44] Rogers EM (2001). The Digital Divide. Convergence.

[45] Jacobs JC, Hu J, Slightam C, Gregory A, Zulman DM (2020). Virtual Savings: Patient-Reported Time and Money Savings from a VA National Telehealth Tablet Initiative. Telemed E-Health.

[46] Hirko KA, Kerver JM, Ford S (2020). Telehealth in response to the COVID-19 pandemic: Implications for rural health disparities. J Am Med Inform Assoc.

[47] Albert NM, Prasun MA (2020). Telemedicine in Heart Failure during COVID-19: Like it, Love It or Lose It?. Heart Lung.

[48] Takahashi EA, Schwamm LH, Adeoye OM (2022). An Overview of Telehealth in the Management of Cardiovascular Disease: A Scientific Statement From the American Heart Association. Circulation.

[49] Enhancing Veterans’ Access to Care through Video Telehealth Tablets QUERI. Available from: https://www.queri.research.va.gov/centers/VideoTablets.pdf

[50] **Zulman DM, Wong EP, Slightam C, et al**. Making connections: nationwide implementation of video telehealth tablets to address access barriers in veterans. JAMIA Open 2019;2(3):323–9. 10.1093/jamiaopen/ooz024 PMC6952023 32766533

[51] Lifeline Support for Affordable Communications [Internet]. Fed. Commun. Comm. 2016 [cited 2023 Mar 28]; Available from: https://www.fcc.gov/lifeline-consumers

[52] Affordable Connectivity Program [Internet]. Fed. Commun. Comm. 2021 [cited 2023 Mar 28]; Available from: https://www.fcc.gov/acp

[53] EveryoneOn [Internet]. EveryoneOn. [cited 2023 Mar 28]; Available from: https://www.everyoneon.org

[54] **Rubenstein LV, Curtis I, Wheat CL, et al**. Learning from national implementation of the Veterans Affairs Clinical Resource Hub (CRH) program for improving access to care: protocol for a six year evaluation. BMC Health Serv Res 2023;23(1):790. 10.1186/s12913-023-09799-5 PMC10367243 37488518

[55] **Gray C, Wray C, Tisdale R, Chaudary C, Slightam C, Zulman D**. Factors Influencing How Providers Assess the Appropriateness of Video Visits: Interview Study With Primary and Specialty Health Care Providers. J Med Internet Res 2022;24(8):e38826. 10.2196/38826 PMC9453588 36001364

