# Supplementary material for: Disparities in Video-Based Primary Care Use Among Veterans with Cardiovascular Disease
Source: J Gen Intern Med. 2024 Jan 22;39(Suppl 1):60–7. doi: 10.1007/s11606-023-08475-y (PMC10937859; doi:10.1007/s11606-023-08475-y)
Supplement: Supplementary file 1 — Supplementary file1 (DOCX 56 KB) [file 11606_2023_8475_MOESM1_ESM.docx]

Online Supplement

Disparities in Video Care Use Among Veterans with Cardiovascular Disease

Supplemental Methods

*Pandemic Year Classification*

We classified 3/16/2019 through 3/15/2020 as pre-pandemic, 3/16/2020 through 3/15/2021 as pandemic year one, and 3/16/2021 through 3/15/2022 as pandemic year two.

*Race/Ethnicity Classification*

Race/ethnicity was defined as non-Hispanic Black, Hispanic, non-Hispanic Other (which included American Indian/Alaska Native, Asian, and Native Hawaiian/Pacific Islander Veterans), Unknown, or White.

*Marital Status Classification*

Marital status was defined as divorced, married, never married, separated, widowed, or unknown.

*Rurality Classification*

Rurality included highly rural, insular (i.e., in a U.S. jurisdiction outside of a state or Federal district, such as Guam), rural, urban, and unknown.

*VHA Enrollment Priority Classification*

VHA’s enrollment priority classification system categorizes VHA patients according to military service-related disability, income, and other factors. Veterans with disabilities related to their military service are assigned the highest priority, and those without service-connected disabilities who earn a higher income receive the lowest priority. In accordance with prior work (2,16) we have condensed these categories to four: high disability, corresponding to enrollment priority groups 1 and 4; low-moderate disability, including priority groups 2, 3, and 6; low-income, including priority group 5; and no disability, wherein patients pay co-pays for VHA care, including priority groups 7-8. Note that Veterans assigned to high- or low-moderate disability groups may also be low-income due to the hierarchical nature of these enrollment groups.

Supplemental Table 1. International Classification of Disease-10 (ICD10) Codes

Cardiomyopathy

I42.X I43

Heart failure

I50 I501 I502 I5020 I5021 I5022 I5023

I503 I5030 I5031 I5032 I5033 I504 I5040

I5041 I5042 I5043 I508 I5081 I50810 I50811

I50812 I50813 I50814 I5082 I5083 I5089 I509

I0981 I110 I130 I132

Hypertension

I10 I11.X I12.X I13.X I15.X I16.X

Supplemental Table 2. Baseline Patient Characteristics and Video Care Use Over Time, Veterans with Hypertension (with or without Heart Failure) (N=3,757,067)^*^

|  | Pre-Pandemic Year | | | | Pandemic Year 1 | | | | Pandemic Year 2 | | | |
| --- | --- | --- | --- | --- | --- | --- | --- | --- | --- | --- | --- | --- |
|  | Total | 0 | 1 | 2+ | Total | 0 | 1 | 2+ | Total | 0 | 1 | 2+ |
| N (%) | 3757067 | 3707444 (98.7) | 36217  (1.0) | 13406  (0.4) | -^†^ | 3162796 (84.2) | 401729  (10.7) | 192542  (5.1) | - | 3274021  (87.1) | 318384  (8.5) | 164662  (4.4) |
|  | | | | | | | | | | | | |
| Baseline Age, years (mean [SD]) | 66.7 (13.0) | 66.7 (13.0) | 64.2 (13.4) | 63.6 (13.2) | - | 67.4 (12.8) | 63.3 (13.2) | 61.3 (13.3) | - | 67.4 (12.7) | 62.2 (13.4) | 59.9 (13.4) |
| Baseline Age, years, categorical |  | | | | | | | | | | | |
| 18-44 | 240119 (6.4) | 235633 (6.4) | 3234 (8.9) | 1252 (9.3) | - | 179220 (5.7) | 38433 (9.6) | 22466 (11.7) | - | 182698 (5.6) | 34700 (10.9) | 22721 (13.8) |
| 45-64 | 1153776 (30.7) | 1135971 (30.6) | 12866 (35.5) | 4939 (36.8) | - | 915696 (29.0) | 154467 (38.5) | 83613 (43.4) | - | 949283 (29.0) | 129753 (40.8) | 74740 (45.4) |
| 65-74 | 1442595 (38.4) | 1424608 (38.4) | 13098 (36.2) | 4889 (36.5) | - | 1237859 (39.1) | 142932 (35.6) | 61804 (32.1) | - | 1286984 (39.3) | 106590 (33.5) | 49021 (29.8) |
| 75+ | 920569 (24.5) | 911224 (24.6) | 7019 (19.4) | 2326 (17.4) | - | 830013 (26.2) | 65897 (16.4) | 24659 (12.8) | - | 855053 (26.1) | 47339 (14.9) | 18177 (11.0) |
| Birth Sex |  | | | | | | | | | | | |
| Female | 210620 (5.6) | 206314 (5.6) | 3024 (8.4) | 1282 (9.6) | - | 155308 (4.9) | 32896 (8.2) | 22416 (11.6) | - | 159842 (4.9) | 29185 (9.2) | 21593 (13.1) |
| Race/  Ethnicity |  | | | | | | | | | | | |
| NH Black | 707737 (18.8) | 699920 (18.9) | 5777 (16.0) | 2040 (15.2) | - | 571395 (18.1) | 87955 (21.9) | 48387 (25.1) | - | 585680 (17.9) | 76643 (24.1) | 45414 (27.6) |
| Hispanic | 206171 (5.5) | 203434 (5.5) | 1999 (5.5) | 738 (5.5) | - | 160652 (5.1) | 28021 (7.0) | 17498 (9.1) | - | 170815 (5.2) | 22362 (7.0) | 12994 (7.9) |
| NH Other | 84920 (2.3) | 83512 (2.3) | 1065 (2.9) | 343 (2.6) | - | 70102 (2.2) | 9845 (2.5) | 4973 (2.6) | - | 71906 (2.2) | 8316 (2.6) | 4698 (2.9) |
| Unknown | 224053 (6.0) | 221378 (6.0) | 1922 (5.3) | 753 (5.6) | - | 193391 (6.1) | 21321 (5.3) | 9341 (4.9) | - | 196686 (6.0) | 18246 (5.7) | 9121 (5.5) |
| White | 2534186 (67.5) | 2499200 (67.4) | 25454 (70.3) | 9532 (71.1) | - | 2167256 (68.5) | 254587 (63.4) | 112343 (58.4) | - | 2248934 (68.7) | 192817 (60.6) | 92435 (56.1) |
| Enrollment Priority |  |  |  |  |  |  |  |  |  |  |  |  |
| High disability | 1265302 (33.7) | 1245251 (33.6) | 14236 (39.3) | 5815 (43.4) | - | 1023746 (32.4) | 157328 (39.2) | 84228 (43.8) | - | 1068311 (32.6) | 125971 (39.6) | 71020 (43.1) |
| Low/  moderate disability | 889442 (23.7) | 877444 (23.7) | 8888 (24.5) | 3110 (23.2) | - | 748307 (23.7) | 97987 (24.4) | 43148 (22.4) | - | 779059 (23.8) | 73957 (23.2) | 36426 (22.1) |
| Low-Income | 742232 (19.8) | 733550 (19.8) | 6262 (17.3) | 2420 (18.1) | - | 645723 (20.4) | 65504 (16.3) | 31005 (16.1) | - | 664433 (20.3) | 52285 (16.4) | 25514 (15.5) |
| No service disability | 706601 (18.8) | 698475 (18.8) | 6226 (17.2) | 1900 (14.2) | - | 613639 (19.4) | 66094 (16.5) | 26868 (14.0) | - | 633308 (19.3) | 50574 (15.9) | 22719 (13.8) |
| Unknown | 153490 (4.1) | 152724 (4.1) | 605 (1.7) | 161 (1.2) | - | 131381 (4.2) | 14816 (3.7) | 7293 (3.8) | - | 128910 (3.9) | 15597 (4.9) | 8983 (5.5) |
| Rurality |  |  |  |  |  |  |  |  |  |  |  |  |
| Highly Rural | 49038 (1.3) | 47550 (1.3) | 1138 (3.1) | 350 (2.6) | 154997 (4.1) | 139609 (4.4) | 11167 (2.8) | 4221 (2.2) | 152945 (4.1) | 140457 (4.3) | 8562 (2.7) | 3926 (2.4) |
| Insular | 3424 (0.1) | 3191 (0.1) | 191 (0.5) | 42 (0.3) | 3473 (0.1) | 3130 (0.1) | 228 (0.1) | 115 (0.1) | 3402 (0.1) | 3090 (0.1) | 214 (0.1) | 98 (0.1) |
| Rural | 1251922 (33.3) | 1229536 (33.2) | 15958 (44.1) | 6428 (48.0) | 1175936 (31.3) | 1030917 (32.6) | 102313 (25.5) | 42706 (22.2) | 1170778 (31.2) | 1052375 (32.1) | 80405 (25.3) | 37998 (23.1) |
| Urban | 2285539 (60.8) | 2261068 (61.0) | 18120 (50.0) | 6351 (47.4) | 2315209 (61.6) | 1892571 (59.8) | 280600 (69.9) | 142038 (73.8) | 2304617 (61.3) | 1960637 (59.9) | 224122 (70.4) | 119858 (72.8) |
| Unknown | 167144 (4.5) | 166099 (4.5) | 810 (2.2) | 235 (1.8) | 107452 (2.9) | 96569 (3.1) | 7421 (1.9) | 3462 (1.8) | 125325 (3.3) | 117462 (3.6) | 5081 (1.6) | 2782 (1.7) |
| Charlson Comorbidity Index, mean (SD) | 1.4  (2.0) | 1.4  (1.9) | 1.5 (1.9) | 1.6 (2.0) | 1.6  (2.1) | 1.6  (2.1) | 1.5 (2.0) | 1.7 (2.2) | 1.5  (2.0) | 1.5  (2.1) | 1.5 (2.0) | 1.6 (2.1) |
| Charlson Comorbidity Index, n (%) |  | | | | | | | | | | | |
| 0 | 1720755 (45.8) | 1699946 (45.9) | 15591 (43.1) | 5218 (38.9) | 1532941 (40.8) | 1290451 (40.8) | 168852 (42.0) | 73638 (38.3) | 1640863 (43.7) | 1437109 (43.9) | 136509 (42.9) | 67245 (40.8) |
| 1 | 808745 (21.5) | 797178 (21.5) | 8405 (23.2) | 3162 (23.6) | 842089 (22.4) | 707054 (22.4) | 91374 (22.8) | 43661 (22.7) | 840272 (22.4) | 727094 (22.2) | 74533 (23.4) | 38645 (23.5) |
| 2+ | 1227567 (32.7) | 1210320 (32.7) | 12221 (33.7) | 5026 (37.5) | 1382037 (36.8) | 1165291 (36.8) | 141503 (35.2) | 75243 (39.1) | 1275932 (34.0) | 1109818 (33.9) | 107342 (33.7) | 58772 (35.7) |

^*^ Year 0 refers to the pre-pandemic year: 3/16/2019 – 3/15/2020; Year 1 refers to pandemic year 1: 3/16/2020 – 3/15/2021; Year 2 refers to pandemic year 2: 3/16/2021 – 3/15/2022

^†^ Cells with total N unchanged from baseline year are filled with “-“ for clarity.

Supplemental Table 3. Baseline Patient Characteristics and Video Care Use Over Time, Veterans with Heart Failure (with or without Hypertension) (N=507,654)^*^

|  | Pre-Pandemic Year | | | | Pandemic Year 1 | | | | Pandemic Year 2 | | | |
| --- | --- | --- | --- | --- | --- | --- | --- | --- | --- | --- | --- | --- |
|  | Total | 0 | 1 | 2+ | Total | 0 | 1 | 2+ | Total | 0 | 1 | 2+ |
| N (%) | 507,654 | 500751 (98.6) | 4849 (1.0) | 2054 (0.4) | -^†^ | 430477 (84.8) | 49421 (9.7) | 27756 (5.5) | - | 448225 (88.3) | 38253 (7.5) | 21176 (4.2) |
|  | | | | | | | | | | | | |
| Baseline Age, years (mean [SD]) | 71.5 (11.1) | 71.5 (11.1) | 70.0 (11.4) | 69.6 (11.2) | - | 72.0 (10.9) | 69.0 (11.7) | 67.8 (11.7) | - | 72.0 (10.9) | 68.2 (11.8) | 66.6 (11.7) |
| Baseline Age, years, categorical |  | | | | | | | | | | | |
| 18-44 | 8746 (1.7) | 8589 (1.7) | 110 (2.3) | 47 (2.3) | - | 6335 (1.5) | 1483 (3.0) | 928 (3.3) | - | 6525 (1.5) | 1338 (3.5) | 883 (4.2) |
| 45-64 | 107035 (21.1) | 105321 (21.0) | 1185 (24.4) | 529 (25.8) | - | 84711 (19.7) | 13500 (27.3) | 8824 (31.8) | - | 88491 (19.7) | 11321 (29.6) | 7223 (34.1) |
| 65-74 | 210367 (41.4) | 207434 (41.4) | 2066 (42.6) | 867 (42.2) | - | 178367 (41.4) | 20679 (41.8) | 11321 (40.8) | - | 186054 (41.5) | 15741 (41.2) | 8572 (40.5) |
| 75+ | 181505 (35.8) | 179406 (35.8) | 1488 (30.7) | 611 (29.8) | - | 161063 (37.4) | 13759 (27.8) | 6683 (24.1) | - | 167154 (37.3) | 9853 (25.8) | 4498 (21.2) |
| Birth Sex |  | | | | | | | | | | | |
| Female | 15894 (3.1) | 15551 (3.1) | 239 (4.9) | 104 (5.1) | - | 11943 (2.8) | 2246 (4.5) | 1705 (6.1) | - | 12487 (2.8) | 1867 (4.9) | 1540 (7.3) |
| Race/  Ethnicity |  | | | | | | | | | | | |
| NH Black | 91249 (17.8) | 90275 (18.0) | 710 (14.6) | 264 (12.9) | - | 75207 (17.5) | 9974 (20.2) | 6068 (21.9) | - | 77577 (17.3) | 8521 (22.3) | 5151 (24.3) |
| Hispanic | 22382 (4.4) | 22083 (4.4) | 199 (4.1) | 100 (4.9) | - | 17714 (4.1) | 2698 (5.5) | 1970 (7.1) | - | 18985 (4.2) | 2103 (5.5) | 1294 (6.1) |
| NH Other | 9225 (1.8) | 9060 (1.8) | 116 (2.4) | 49 (2.4) | - | 7699 (1.8) | 975 (2.0) | 551 (2.0) | - | 8027 (1.8) | 740 (1.9) | 458 (2.2) |
| Unknown | 27205 (5.4) | 26827 (5.4) | 264 (5.4) | 114 (5.6) | - | 23564 (5.5) | 2400 (4.9) | 1241 (4.5) | - | 24185 (5.4) | 1996 (5.2) | 1024 (4.8) |
| White | 357593 (70.4) | 352506 (70.4) | 3560 (73.4) | 1527 (74.3) | - | 306293 (71.2) | 33374 (67.5) | 17926 (64.6) | - | 319451 (71.3) | 24893 (65.1) | 13249 (62.6) |
| Enrollment Priority |  | | | | | | | | | | | |
| High disability | 184964 (36.4) | 182141 (36.4) | 1965 (40.5) | 858 (41.8) | - | 153139 (35.6) | 19657 (39.8) | 12168 (43.8) | - | 160518 (35.8) | 15355 (40.1) | 9091 (42.9) |
| Low/  moderate disability | 92356 (18.2) | 91076 (18.2) | 910 (18.8) | 370 (18.0) | - | 77915 (18.1) | 9447 (19.1) | 4994 (18.0) | - | 81299 (18.1) | 7106 (18.6) | 3951 (18.7) |
| Low-Income | 132168 (26.0) | 130509 (26.1) | 1139 (23.5) | 520 (25.3) | - | 114669 (26.6) | 11289 (22.8) | 6210 (22.4) | - | 118706 (26.5) | 8799 (23.0) | 4663 (22.0) |
| No service disability | 84844 (16.7) | 83776 (16.7) | 781 (16.1) | 287 (14.0) | - | 73376 (17.1) | 7748 (15.7) | 3720 (13.4) | - | 76278 (17.0) | 5739 (15.0) | 2827 (13.4) |
| Unknown | 13322 (2.6) | 13249 (2.7) | 54 (1.1) | 19 (0.9) | - | 11378 (2.6) | 1280 (2.6) | 664 (2.4) | - | 11424 (2.6) | 1254 (3.3) | 644 (3.0) |
| Rurality |  | | | | | | | | | | | |
| Highly Rural | 6883 (1.4) | 6688 (1.3) | 138 (2.9) | 57 (2.8) | 21223 (4.2) | 19198 (4.5) | 1409 (2.9) | 616 (2.2) | 20198 (4.0) | 18615 (4.2) | 1042 (2.7) | 541 (2.6) |
| Insular | 328 (0.1) | 298 (0.1) | 23 (0.5) | 7 (0.3) | 322 (0.1) | 292 (0.1) | 16 (0.0) | 14 (0.1) | 303 (0.1) | 276 (0.1) | 18 (0.1) | 9  (0.0) |
| Rural | 169699 (33.4) | 166658 (33.3) | 2086 (43.0) | 955 (46.5) | 156101 (30.8) | 137340 (31.9) | 12472 (25.2) | 6289 (22.7) | 150209 (29.6) | 135676 (30.3) | 9671 (25.3) | 4862 (23.0) |
| Urban | 316110 (62.3) | 312568 (62.4) | 2532 (52.2) | 1010 (49.2) | 316479 (62.3) | 261069 (60.7) | 34868 (70.6) | 20542 (74.0) | 305356 (60.2) | 262677 (58.6) | 27117 (70.9) | 15562 (73.5) |
| Unknown | 14634 (2.9) | 14539 (2.9) | 70 (1.4) | 25 (1.22) | 13529 (2.7) | 12578 (2.9) | 656 (1.3) | 295 (1.1) | 31588 (6.2) | 30981 (6.9) | 405 (1.1) | 202 (1.0) |
| Charlson Comorbidity Index, mean (SD) | 2.9  (2.6) | 2.9  (2.6) | 3.0 (2.6) | 3.2 (2.7) | 3.5  (2.8) | 3.5  (2.9) | 3.3 (2.7) | 3.7 (2.8) | 3.1  (2.8) | 3.1  (2.9) | 3.3 (2.7) | 3.5 (2.8) |
| Charlson Comorbidity Index, n (%) |  | | | | | | | | | | | |
| 0 | 107121 (21.1) | 105939 (21.2) | 873 (18) | 309 (15.0) | 65966 (13.0) | 55973 (13.0) | 6787 (13.7) | 3206 (11.6) | 96986 (19.1) | 89283 (19.9) | 5246 (13.7) | 2457 (11.6) |
| 1 | 86269 (17.0) | 85062 (17.0) | 877 (18.1) | 330 (16.1) | 78775 (15.5) | 66793 (15.5) | 7930 (16.1) | 4052 (14.6) | 79043 (15.6) | 69342 (15.5) | 6451 (16.9) | 3250 (15.4) |
| 2+ | 314264 (61.9) | 309750 (61.9) | 3099 (63.9) | 1415 (68.9) | 362913 (71.5) | 307711 (71.5) | 34704 (70.2) | 20498 (73.9) | 331625 (65.3) | 289600 (64.6) | 26556 (69.4) | 15469 (73.1) |

^*^ Year 0 refers to the pre-pandemic year: 3/16/2019 – 3/15/2020; Year 1 refers to pandemic year 1: 3/16/2020 – 3/15/2021; Year 2 refers to pandemic year 2: 3/16/2021 – 3/15/2022

^†^ Cells with total N unchanged from baseline year are filled with “-“ for clarity.

Supplemental Table 4. Full Results of Main Statistical Model: Adjusted Odds of Ever Using Video Care Among 3,807,820 Veterans with Hypertension, Heart Failure, or Both

|  | **Main Model^*^** |
| --- | --- |
| **Characteristic** | **Adjusted Odds Ratio (95% Confidence Interval)** |
| **Baseline Age category** |  |
| 18-44 | (ref) |
| 45-64 | 0.74 (0.74, 0.75) |
| 65-74 | 0.49 (0.48, 0.50) |
| 75+ | 0.38 (0.37, 0.38) |
| **Birth Sex** |  |
| Female | (ref) |
| Male | 0.73 (0.72, 0.74) |
| **Baseline Race/Ethnicity** |  |
| Non-Hispanic White | (ref) |
| Non-Hispanic Black | 1.04 (1.03, 1.05) |
| Hispanic | 1.17 (1.15, 1.18) |
| Non-Hispanic Other Race | 1.05 (1.03, 1.07) |
| Unknown Race/Ethnicity | 1.00 (0.99, 1.01) |
| **Rurality** |  |
| Urban | (ref) |
| Rural or highly rural | 0.71 (0.70, 0.71) |
| **Enrollment Priority** |  |
| High disability | (ref) |
| Low/moderate disability | 0.92 (0.92, 0.93) |
| Low income | 0.79 (0.78, 0.80) |
| No service disability | 0.87 (0.87, 0.88) |
| **Marital Status** |  |
| Married | (ref) |
| Divorced or widowed | 0.89 (0.88, 0.89) |
| Never married or single | 0.83 (0.82, 0.84) |
| **Heart Failure** |  |
| No heart failure diagnosis | (ref) |
| Heart failure diagnosis | 1.05 (1.04, 1.06) |
| **Charlson Comorbidity Index** |  |
| 0 | (ref) |
| 1 | 0.99 (0.98, 1.00) |
| 2+ | 1.03 (1.02, 1.04) |
| **Covid Year** |  |
| 0 | (ref) |
| 1 | 15.3 (15.1, 15.4) |
| 2+ | 11.5 (11.3, 11.6) |

^*^ Mixed logistic regression model with two-level clustering at patient and VA facility levels

Supplemental Table 5. Adjusted Odds Ratios for Video Care Use: Sensitivity Analyses Among 3,807,820 Veterans with Hypertension, Heart Failure, or Both Including Interaction Terms for Sociodemographic Characteristics

| **Interaction Included** | **Race x rural** | **Sex x age** | **Rural x age** | **Race x sex** | **Race x age** | **Rural x sex** |
| --- | --- | --- | --- | --- | --- | --- |
| **Characteristic** | **Adjusted Odds Ratio (95% Confidence Interval)** | | | | | |
| **Baseline Age Category** |  | | | | | |
| 18-44 | (ref) | (ref) | (ref) | (ref) | (ref) | (ref) |
| 45-64 | 0.74 (0.74, 0.75) | 0.74 (0.74, 0.75) | 0.75 (0.74, 0.76) | 0.74 (0.74, 0.75) | 0.76 (0.74, 0.77) | 0.74 (0.74, 0.75) |
| 65-74 | 0.49 (0.48, 0.50) | 0.49 (0.48, 0.50) | 0.51 (0.50, 0.51) | 0.49 (0.48, 0.50) | 0.51 (0.50, 0.51) | 0.49 (0.48, 0.50) |
| 75+ | 0.38 (0.37, 0.38) | 0.38 (0.37, 0.38) | 0.39 (0.38, 0.39) | 0.38 (0.37, 0.38) | 0.39 (0.38, 0.39) | 0.38 (0.37, 0.38) |
| **Birth Sex** |  | | | | | |
| Female | (ref) | (ref) | (ref) | (ref) | (ref) | (ref) |
| Male | 0.73 (0.72, 0.74) | 0.78 (0.76, 0.81) | 0.73 (0.72, 0.73) | 0.75 (0.74, 0.76) | 0.73 (0.72, 0.74) | 0.74 (0.73, 0.75) |
| **Sex x Age** |  | | | | | |
| 18-44 x Female | N/A | (ref) | N/A | N/A | N/A | N/A |
| 45-64 x Male | N/A | 0.92 (0.89, 0.95) | N/A | N/A | N/A | N/A |
| 65-74 x Male | N/A | 0.87 (0.83, 0.91) | N/A | N/A | N/A | N/A |
| 75+ x Male | N/A | 1.00 (0.95, 1.07) | N/A | N/A | N/A | N/A |
| **Race/Ethnicity** |  | | | | | |
| Non-Hispanic White | (ref) | (ref) | (ref) | (ref) | (ref) | (ref) |
| Non-Hispanic Black | 1.04 (1.03, 1.05) | 1.04 (1.03, 1.05) | 1.04 (1.04, 1.05) | 1.12 (1.09, 1.14) | 1.10 (1.08, 1.13) | 1.04 (1.03, 1.05) |
| Hispanic | 1.14 (1.12, 1.15) | 1.17 (1.15, 1.18) | 1.17 (1.15, 1.18) | 1.13 (1.08, 1.19) | 1.20 (1.16, 1.24) | 1.17 (1.15, 1.18) |
| Non-Hispanic Other Race | 1.08 (1.05, 1.10) | 1.05 (1.03, 1.07) | 1.05 (1.03, 1.07) | 0.97 (0.91, 1.04) | 1.06 (1.00, 1.12) | 1.05 (1.03, 1.07) |
| Unknown Race/Ethnicity | 1.00 (0.99, 1.02) | 1.00 (0.99, 1.01) | 1.00 (0.99, 1.02) | 1.03 (0.98, 1.08) | 1.10 (1.04, 1.15) | 1.00 (0.99, 1.01) |
| **Race x Sex** |  | | | | | |
| Non-Hispanic White x Female | N/A | N/A | N/A | (ref) | N/A | N/A |
| Non-Hispanic Black x Male | N/A | N/A | N/A | 0.92 (0.90, 0.94) | N/A | N/A |
| Hispanic x Male | N/A | N/A | N/A | 1.03 (0.98, 1.09) | N/A | N/A |
| Non-Hispanic Other Race x Male | N/A | N/A | N/A | 1.08 (1.01, 1.16) | N/A | N/A |
| Unknown Race/ Ethnicity x Male | N/A | N/A | N/A | 0.97 (0.92, 1.02) | N/A | N/A |
| **Race x Age** |  | | | | | |
| Non-Hispanic White x 18-44 | N/A | N/A | N/A | N/A | (ref) | N/A |
| Non-Hispanic Black x 45-64 | N/A | N/A | N/A | N/A | 0.95 (0.93, 0.98) | N/A |
| Non-Hispanic Black x 65-74 | N/A | N/A | N/A | N/A | 0.91 (0.88, 0.93) | N/A |
| Non-Hispanic Black x 75+ | N/A | N/A | N/A | N/A | 0.99 (0.96, 1.03) | N/A |
| Hispanic x 45-64 | N/A | N/A | N/A | N/A | 1.01 (0.97, 1.05) | N/A |
| Hispanic x 65-74 | N/A | N/A | N/A | N/A | 0.92 (0.89, 0.96) | N/A |
| Hispanic x 75+ | N/A | N/A | N/A | N/A | 0.99 (0.95, 1.04) | N/A |
| Non-Hispanic Other Race x 45-64 | N/A | N/A | N/A | N/A | 1.00 (0.94, 1.06) | N/A |
| Non-Hispanic Other Race x 65-74 | N/A | N/A | N/A | N/A | 0.96 (0.90, 1.03) | N/A |
| Non-Hispanic Other Race x 75+ | N/A | N/A | N/A | N/A | 1.01 (0.94, 1.09) | N/A |
| Unknown Race/ Ethnicity x 45-64 | N/A | N/A | N/A | N/A | 0.94 (0.89, 0.99) | N/A |
| Unknown Race/ Ethnicity x 65-74 | N/A | N/A | N/A | N/A | 0.91 (0.86, 0.96) | N/A |
| Unknown Race/ Ethnicity x 75+ | N/A | N/A | N/A | N/A | 0.86 (0.91, 0.91) | N/A |
| **Rurality** |  | | | | | |
| Urban | (ref) | (ref) | (ref) | (ref) | (ref) | (ref) |
| Rural or highly rural | 0.70 (0.70, 0.71) | 0.71 (0.70, 0.71) | 0.77 (0.75, 0.79) | 0.71 (0.70, 0.71) | 0.71 (0.70, 0.71) | 0.75 (0.73, 0.77) |
| **Rurality x Age** |  | | | | | |
| 18-44 x Urban | N/A | N/A | (ref) | N/A | N/A | N/A |
| 45-64 x Rural | N/A | N/A | 0.94 (0.93, 0.97) | N/A | N/A | N/A |
| 65-74 x Rural | N/A | N/A | 0.89 (0.88, 0.89) | N/A | N/A | N/A |
| 75+ x Rural | N/A | N/A | 0.91 (0.89, 0.94) | N/A | N/A | N/A |
| **Rurality x Sex** |  | | | | | |
| Urban x Female | N/A | N/A | N/A | N/A | N/A | (ref) |
| Rural x Male | N/A | N/A | N/A | N/A | N/A | 0.94 (0.92, 0.97) |
| **Race x Rurality** |  | | | | | |
| Non-Hispanic White x Urban | (ref) | N/A | N/A | N/A | N/A | N/A |
| Non-Hispanic Black x Rural | 0.99 (0.97, 1.01) | N/A | N/A | N/A | N/A | N/A |
| Hispanic x Rural | 1.18 (1.15, 1.22) | N/A | N/A | N/A | N/A | N/A |
| Non-Hispanic Other Race x Rural | 0.88 (0.85, 0.93) | N/A | N/A | N/A | N/A | N/A |
| Unknown Race/ Ethnicity x Rural | 0.98 (0.95, 1.00) | N/A | N/A | N/A | N/A | N/A |
| **Enrollment Priority** |  | | | | | |
| High disability | (ref) | (ref) | (ref) | (ref) | (ref) | (ref) |
| Low/moderate disability | 0.92 (0.91, 0.93) | 0.92 (0.92, 0.93) | 0.92 (0.91, 0.93) | 0.92 (0.92, 0.93) | 0.92 (0.91, 0.93) | 0.92 (0.92, 0.93) |
| Low income | 0.79 (0.78, 0.80) | 0.79 (0.78, 0.80) | 0.79 (0.78, 0.80) | 0.79 (0.78, 0.80) | 0.79 (0.78, 0.80) | 0.79 (0.78, 0.80) |
| No service disability | 0.87 (0.86, 0.88) | 0.87 (0.87, 0.88) | 0.87 (0.86, 0.88) | 0.87 (0.87, 0.88) | 0.87 (0.87, 0.88) | 0.87 (0.87, 0.88) |
| **Marital Status** |  | | | | | |
| Married | (ref) | (ref) | (ref) | (ref) | (ref) | (ref) |
| Divorced or widowed | 0.89 (0.88, 0.89) | 0.89 (0.88, 0.89) | 0.89 (0.88, 0.90) | 0.89 (0.88, 0.89) | 0.89 (0.88, 0.89) | 0.89 (0.88, 0.89) |
| Never married or single | 0.83 (0.82, 0.84) | 0.83 (0.82, 0.84) | 0.83 (0.82, 0.84) | 0.83 (0.82, 0.84) | 0.83 (0.82, 0.84) | 0.83 (0.82, 0.84) |
| **Heart Failure** |  | | | | | |
| No heart failure diagnosis | (ref) | (ref) | (ref) | (ref) | (ref) | (ref) |
| Heart failure diagnosis | 1.05 (1.04, 1.06) | 1.05 (1.04, 1.06) | 1.05 (1.04, 1.06) | 1.05 (1.04, 1.06) | 1.05 (1.04, 1.06) | 1.05 (1.04, 1.06) |
| **Charlson Comorbidity Index** |  | | | | | |
| 0 | (ref) | (ref) | (ref) | (ref) | (ref) | (ref) |
| 1 | 0.99 (0.98, 1.00) | 0.99 (0.98, 1.00) | 0.99 (0.98, 1.00) | 0.99 (0.98, 1.00) | 0.99 (0.98, 1.00) | 0.99 (0.98, 1.00) |
| 2+ | 1.03 (1.02, 1.04) | 1.03 (1.02, 1.04) | 1.03 (1.02, 1.04) | 1.03 (1.02, 1.04) | 1.03 (1.02, 1.04) | 1.03 (1.02, 1.04) |
| **Covid Year** |  | | | | | |
| 0 | (ref) | (ref) | (ref) | (ref) | (ref) | (ref) |
| 1 | 15.2 (15.1, 15.4) | 15.2 (15.1, 15.4) | 15.2 (15.1, 15.4) | 15.2 (15.1, 15.4) | 15.2 (15.1, 15.4) | 15.2 (15.1, 15.4) |
| 2+ | 11.5 (11.3, 11.6) | 11.5 (11.3, 11.6) | 11.5 (11.3, 11.6) | 11.5 (11.3, 11.6) | 11.5 (11.3, 11.6) | 11.5 (11.3, 11.6) |

Supplemental Table 6. Adjusted Odds Ratios for Video Care Use: Sensitivity Analyses Among 3,807,820 Veterans with Hypertension, Heart Failure, or Both Including Additional Sociodemographic Characteristics

|  | **Main Model^*^** |
| --- | --- |
| **Characteristic** | **Adjusted Odds Ratio (95% Confidence Interval)** |
| **Baseline Age category** |  |
| 18-44 | (ref) |
| 45-64 | 0.74 (0.74, 0.75) |
| 65-74 | 0.49 (0.49, 0.50) |
| 75+ | 0.38 (0.38, 0.39) |
| **Birth Sex** |  |
| Female | (ref) |
| Male | 0.73 (0.72, 0.74) |
| **Baseline Race/Ethnicity** |  |
| Non-Hispanic White | (ref) |
| Non-Hispanic Black | 1.00 (1.00, 1.01) |
| Hispanic | 1.11 (1.10, 1.13) |
| Non-Hispanic Other Race | 1.04 (1.02, 1.06) |
| Unknown Race/Ethnicity | 0.98 (0.96, 0.99) |
| **Rurality** |  |
| Urban | (ref) |
| Rural or highly rural | 0.78 (0.77, 0.79) |
| **Enrollment Priority** |  |
| High disability | (ref) |
| Low/moderate disability | 0.93 (0.92, 0.93) |
| Low income | 0.79 (0.78, 0.80) |
| No service disability | 0.88 (0.87, 0.89) |
| **Marital Status** |  |
| Married | (ref) |
| Divorced or widowed | 0.89 (0.88, 0.89) |
| Never married or single | 0.83 (0.82, 0.84) |
| **Heart Failure** |  |
| No heart failure diagnosis | (ref) |
| Heart failure diagnosis | 1.04 (1.04, 1.05) |
| **Charlson Comorbidity Index** |  |
| 0 | (ref) |
| 1 | 0.99 (0.98, 1.00) |
| 2+ | 1.03 (1.02, 1.03) |
| **Covid Year** |  |
| 0 | (ref) |
| 1 | 15.3 (15.1, 15.5) |
| 2+ | 11.5 (11.4, 11.6) |
| **Drive Distance** |  |
| ≤40 miles | (ref) |
| >40 miles | 0.91 (0.90, 0.92) |
| **Patient Broadband Access** |  |
| No broadband access in patient’s residence census block | (ref) |
| Broadband access in patient’s resident census block | 1.17 (1.16,1.19) |
| **Complexity of Patient’s Primary VA Facility** |  |
| High | (ref) |
| Low or medium | 0.89 (0.89, 0.89) |
